# Middle East Respiratory Syndrome Coronavirus (MERS-CoV) is Not Circulating Among Hajj Pilgrims

**DOI:** 10.1007/s44197-023-00128-x

**Published:** 2023-06-16

**Authors:** Jaffar A. Al-Tawfiq, Ziad A. Memish

**Affiliations:** 1 Specialty Internal Medicine and Quality Department, Johns Hopkins Aramco Healthcare, Dhahran, Saudi Arabia; 2 Infectious Disease Division, Department of Medicine, Indiana University School of Medicine, Indianapolis, IN USA; 3 Infectious Disease Division, Department of Medicine, Johns Hopkins University School of Medicine, Baltimore, MD USA; 4 Research and Innovation Center, King Saud Medical City, Ministry of Health, Jeddah, Saudi Arabia; 5 College of Medicine, Alfaisal University, Riyadh, Saudi Arabia; 6 Hubert Department of Global Health, Rollins School of Public Health, Emory University, Atlanta, GA USA; 7 Eminent Scientist of Kyung Hee University, Seoul, South Korea

## Abstract

Since the emergence of the middle east respiratory syndrome coronavirus (MERS-CoV) 2012, the virus had caused multiple healthcare-associated outbreaks. The initial 2012 Hajj season started few weeks after the first case of MERS-CoV, but there were no reported cases among pilgrims in 2012. Since then, there had been multiple studies examining the prevalence of MERS-CoV among Hajj pilgrims. Subsequently, multiple studies utilized screening of pilgrims for MERS-CoV and > 10,000 pilgrims were screened with no identifiable cases of MERS.

The Middle East Respiratory Syndrome Coronavirus (MERS-CoV) was first identified in a patient in Saudi Arabia in 2012 [1]. The MERS-CoV is a large, enveloped, positive-strand RNA virus. Human coronaviruses fall into either the alpha or beta genera of the coronavirus, which is divided into four subgenera (alpha, beta, delta, and gamma). Since the 1960s, it has been recognized that four coronaviruses (HCoV 229E, NL63, OC43, and HKU1) are responsible for the common cold and gastrointestinal problems in people. These viruses are spread by wild animals, such as mice, bats, birds, and livestock. The MERS-CoV is a lineage C betacoronavirus. MERS-CoV is still a high-threat pathogen that the WHO has classified as a priority pathogen, because it produces a serious illness with a high fatality rate, the potential for an epidemic, and no available medical treatments. MERS-CoV has been found in dromedaries in a number of Middle Eastern, African, and South Asian nations. There are many different clinical manifestations of MERS-CoV-2, but the respiratory system is most frequently impacted. According to the World Health Organization (WHO), 2600 laboratory-confirmed cases of MERS-CoV infection have been reported between 2012 and October 17, 2022, with the Kingdom of Saudi Arabia reporting 84% (2193/2600) of those cases.

Healthcare-associated infections are the distinguishing feature of MERS-CoV infection, despite the description of several family clusters of the virus. There had been several nosocomial outbreaks of human-to-human transmission, with the biggest outbreak taking place in South Korea in 2015 [2] and in Riyadh and Jeddah in 2014 [3]. Even in healthcare settings, there does not seem to be a sustained human-to-human transmission of MERS-CoV, despite the virus's known capacity to spread from one person to another. This is most likely caused by the MERS-CoV’s 0.8–1.3 reproduction number, which is relatively low [4]. It was estimated that the South Korean MERS outbreak had a modest reproduction rate of 1% [5]. However, in few MERS epidemics in Saudi Arabia and South Korea, the reproduction rate was predicted to be as high as 2–5% [6]. The higher reproduction estimates were likely caused by insufficient infection control efforts, and as detection and prevention techniques improved over time, the estimates decreased. Multiple previous studies were conducted among pilgrims and none showed circulation of MERS-CoV among pilgrims. In addition, there were no cases linked to the Hajj. The initial 2012 Hajj season started few weeks after the first case of MERS-CoV, but there were no reported cases among pilgrims in 2012 [7]. Subsequently, multiple systematic screening of pilgrims for MERS-CoV was done and included > 10,000 pilgrims and none of these studies revealed MERS-CoV [8–22] (Table 1). A recent study of the evaluation of admitted patients peri-Hajj season in 2022 showed no MERS-CoV among pilgrims or residents and only one case was diagnosed among a Saudi citizen [23].

**Table 1:** Systematic screening of MERS-CoV utilizing PCR among pilgrims

Reference	Year of the study	Study population	Method	Number of positive/number tested (% positive)
[8]	2012	Symptomatic French	Nasopharyngeal swab	0/300 (0)
[9]	2012	French cohort	Nasopharyngeal swab	0/154 (0)
[22]	2013	Multiple nationalities	Nasopharyngeal swab	0/3210 (0) pre-Hajj; 0/2025 (0) post-Hajj
[10]	2013–2015	Symptomatic British Pilgrims	Upper and lower respiratory tract	0/202 (0)
[11]	2013–15	Ill French travelers	Not indicated	0/33 (0)
[12]	2013–2015	Chinese	Lower respiratory tract sputum, washes, and upper respiratory tract oropharyngeal swab	0/847 (0)
[13]	2015	Jordanian	Nasopharyngeal and oropharyngeal	0/125 (0)
[14]	2014–2015	Kashmir, north India	Nasopharyngeal and throat swabs	0/300 (0)
[15]	2013	Pilgrims from Saudi Arabia, Australia and Qatar	Nasal swabs	0/1038 (0)
[16]	2013	Admitted Pilgrims with Pneumonia	Sputum	0/38 (0)
[17]	2014	Symptomatic pilgrims returning to Austria	sputum, throat swab, or bronchoalveolar lavage	0/7 (0)
[18]	2012–2015	Egyptian	Nasopharyngeal and oropharyngeal swabs	0/484 (0)
[19]	2013	Adult African Hajj pilgrims returning to Ghana, West Africa	Nasopharyngeal swab	0/839 (0)
[20]	2013	Departing Pilgrims, paired and non-paired cohort	Nasal swabs	0/692 (0) (paired cohort); 0/514 (0) (non-paired arriving cohorts); 0/470 (0) non-paired departing cohort
[21]	2013	French pilgrims	Nasal and throat swab	0/129 (0)

In a recent study by Johari et al., authors studied the seroprevalence of anti-MERS-CoV antibodies among Malaysian pilgrims [24]. In the study, a fourfold increase in the anti-MERS-CoV IgG by fourfold between paired pre/post-Hajj was taken as an indication of the acquisition of MERS-CoV during the Hajj in 12 participants. It is important to note that none of the 12 ELISA-positive individuals had anti-MERS neutralizing antibodies. The authors concluded that 0.6% of the Hajj pilgrims returning from the Middle East had developed MERS-CoV serology [24], based on the MERCURIAL study protocol.

The study had significant limitations, first about 38% of the participants being lost for follow up or excluded from the study and this is a high rate that possibly affects the interpretation of the data. Second, of the finally included individuals, 50 (2.5%) and 61 (3.1%) had positive IgG pre- and post-Hajj and 12 (0.6%) had a fourfold increase in the IgG titer. However, none of the individuals were tested for MERS-CoV by PCR, and more importantly none of the positive cases (pre- or post-Hajj) were tested for MERS-CoV by PCR. Of the samples collected in 2018, 20 of 752 pre-Hajj samples (2.7%, 95% CI 0.36–0.90) and 24 of 752 post-Hajj samples (3.2%, 95% CI 2.07–4.58) test positive for MERS-CoV antibodies by ELISA. Of all the samples, the presence of 2.5% positive IgG before the Hajj raises concerns about the interpretation of the data. If the presence of anti-MERS-CoV IgG is an indication of a previous infection, then certainly this raises the question about the prevalence of MERS-CoV among the population of Malaysia. This particular point had not been highlighted or discussed further. Moreover, there had been no reported cases of MERS-CoV from that country. Although, the anti-MERS-CoV IgG is a very sensitive test, it is not very specific. A confirmation of the diagnosis of MERS-CoV requires PCR tests or the confirmation of the presence of anti-MERS-CoV by neutralizing tests or plaque-reduction neutralization tests [25]. None of the 12 participants in the study by Johari et al. had positive neutralizing antibodies, indicating that the result of the anti-MERS IgG to be a false positive. It is a real possibility that the 0.6% seroconversion among returning pilgrims be secondary to other concomitant viral illness (false positive). In addition, it could be argued that the positive antibodies among returning pilgrims could be due to early mild or asymptomatic MERS-CoV infection acquired prior to travel to the Hajj pilgrimage and antibodies developed only after travel. The authors argued that the absence of neutralizing antibodies may indicate only mild subclinical or asymptomatic MERS-CoV infections with subsequent temporary or weak neutralizing antibody responses [24]. Another valid argument is that the absence of neutralizing antibodies indicates that antibodies detected by ELISA is not true positive. In a previous study, anti-MERS-CoV IgG antibodies detected by recombinant immunofluorescence assay (rIFA) is ten times more than that with rELISA [26].

Thus, we find that the conclusion of the study by Johari et al. [24] to be inaccurate and that the study actually did not show any evidence of MERS-CoV acquisition by pilgrims during the studied period from 2016 to 2018 and no spillover infections among the Hajj pilgrims. The epidemiology of MERS-CoV and pattern of transmission had identified three patterns of transmission. The main pattern of transmission of MERS-CoV is nosocomial transmission and this had been well studied previously. There had been limited intra-family transmission, and thus, the absence of transmissions in the community is consistent with the disease epidemiology and is not puzzling at all.

## Data Availability

Not applicable.

## References

[1] Zaki AM, van Boheemen S, Bestebroer TM, Osterhaus ADME, Fouchier RAM (2012). Isolation of a novel coronavirus from a man with pneumonia in Saudi Arabia. N Engl J Med.

[2] Al-Tawfiq JA, Memish ZA (2016). Drivers of MERS-CoV transmission: what do we know?. Expert Rev Respir Med.

[3] Drosten C, Muth D, Corman VM, Hussain R, Al Masri M, HajOmar W (2015). An observational, laboratory-based study of outbreaks of middle east respiratory syndrome coronavirus in Jeddah and Riyadh, Kingdom of Saudi Arabia, 2014. Clin Infect Dis.

[4] Cauchemez S, Fraser C, Van Kerkhove MD, Donnelly CA, Riley S, Rambaut A (2014). Middle East respiratory syndrome coronavirus: quantification of the extent of the epidemic, surveillance biases, and transmissibility. Lancet Infect Dis.

[5] Chowell G, Abdirizak F, Lee S, Lee J, Jung E, Nishiura H (2015). Transmission characteristics of MERS and SARS in the healthcare setting: a comparative study. BMC Med.

[6] Choi S, Jung E, Choi BY, Hur YJ, Ki M (2017). High reproduction number of Middle East respiratory syndrome coronavirus in nosocomial outbreaks: mathematical modelling in Saudi Arabia and South Korea. J Hosp Infect.

[7] Al-Tawfiq JA, Memish ZA (2013). Antibiotic susceptibility and treatment of brucellosis. Recent Pat Antiinfect Drug Discov.

[8] Al-Tawfiq JA, Smallwood CAH, Arbuthnott KG, Malik MSK, Barbeschi M, Memish ZA (2013). Emerging respiratory and novel coronavirus 2012 infections and mass gatherings. East Mediterr Heal J.

[9] Gautret P, Charrel R, Belhouchat K, Drali T, Benkouiten S, Nougairede A (2013). Lack of nasal carriage of novel corona virus (HCoV-EMC) in French Hajj pilgrims returning from the Hajj 2012, despite a high rate of respiratory symptoms. Clin Microbiol Infect.

[10] Atabani SF, Wilson S, Overton-Lewis C, Workman J, Kidd IM, Petersen E (2016). Active screening and surveillance in the United Kingdom for Middle East respiratory syndrome coronavirus in returning travellers and pilgrims from the Middle East: a prospective descriptive study for the period 2013–2015. Int J Infect Dis.

[11] Griffiths K, Charrel R, Lagier J-C, Nougairede A, Simon F, Parola P (2016). Infections in symptomatic travelers returning from the Arabian peninsula to France: a retrospective cross-sectional study. Travel Med Infect Dis.

[12] Ma X, Liu F, Liu L, Zhang L, Lu M, Abudukadeer A (2017). No MERS-CoV but positive influenza viruses in returning Hajj pilgrims, China, 2013–2015. BMC Infect Dis.

[13] Al-Abdallat MM, Rha B, Alqasrawi S, Payne DC, Iblan I, Binder AM (2017). Acute respiratory infections among returning Hajj pilgrims—Jordan, 2014. J Clin Virol.

[14] Koul PA, Mir H, Saha S, Chadha MS, Potdar V, Widdowson M-A (2017). Influenza not MERS CoV among returning Hajj and Umrah pilgrims with respiratory illness, Kashmir, north India, 2014–15. Travel Med Infect Dis.

[15] Barasheed O, Rashid H, Alfelali M, Tashani M, Azeem M, Bokhary H (2014). Viral respiratory infections among Hajj pilgrims in 2013. Virol Sin.

[16] Memish ZA, Almasri M, Turkestani A, Al-Shangiti AM, Yezli S (2014). Etiology of severe community-acquired pneumonia during the 2013 Hajj-part of the MERS-CoV surveillance program. Int J Infect Dis.

[17] Aberle JH, Popow-Kraupp T, Kreidl P, Laferl H, Heinz FX, Aberle SW (2015). Influenza A and B Viruses but Not MERS-CoV in Hajj Pilgrims, Austria, 2014. Emerg Infect Dis.

[18] Refaey S, Amin MM, Roguski K, Azziz-Baumgartner E, Uyeki TM, Labib M (2017). Cross-sectional survey and surveillance for influenza viruses and MERS-CoV among Egyptian pilgrims returning from Hajj during 2012–2015. Influenza Other Respi Viruses.

[19] Annan A, Owusu M, Marfo KS, Larbi R, Sarpong FN, Adu-Sarkodie Y (2015). High prevalence of common respiratory viruses and no evidence of Middle East respiratory syndrome coronavirus in Hajj pilgrims returning to Ghana, 2013. Trop Med Int Heal.

[20] Memish ZA, Assiri A, Turkestani A, Yezli S, Al Masri M, Charrel R (2015). Mass gathering and globalization of respiratory pathogens during the 2013 Hajj. Clin Microbiol Infect.

[21] Benkouiten S, Charrel R, Belhouchat K, Drali T, Nougairede A, Salez N (2014). Respiratory viruses and bacteria among pilgrims during the 2013 Hajj. Emerg Infect Dis.

[22] Memish ZA, Assiri A, Almasri M, Alhakeem RF, Turkestani A, Al Rabeeah AA (2014). Prevalence of MERS-CoV nasal carriage and compliance with the Saudi health recommendations among pilgrims attending the 2013 Hajj. J Infect Dis.

[23] Assiri AM, Alsuraihi H, Alshahrani AMM, Alzaid SZ, Albarraq AM, Asiri S, et al (2023) Viral Etiology of severe acute respiratory illness among admitted patients during the 2022 peri-Hajj season. IJID Reg. 10.1016/J.IJREGI.2023.05.004 10.1016/j.ijregi.2023.05.004 PMC10423662 37583481

[24] Johari J, Hontz RD, Pike BL, Husain T, Rusli N, Mohd-Zain R (2023). MERS-CoV Seroconversion amongst Malaysian Hajj Pilgrims Returning from the Middle East, 2016–2018: Results from the MERCURIAL Multiyear Prospective Cohort Study. Emerg Microbes Infect.

[25] Müller MA, Meyer B, Corman VM, Al-Masri M, Turkestani A, Ritz D (2015). Presence of Middle East respiratory syndrome coronavirus antibodies in Saudi Arabia: a nationwide, cross-sectional, serological study. Lancet Infect Dis.

[26] Drosten C, Meyer B, Müller MA, Corman VM, Al-Masri M, Hossain R (2014). Transmission of MERS-Coronavirus in Household Contacts. N Engl J Med.

